# Disorders of Calcium and Phosphorus Metabolism and the Proteomics/Metabolomics-Based Research

**DOI:** 10.3389/fcell.2020.576110

**Published:** 2020-09-10

**Authors:** Meiheng Sun, Xiaoqiu Wu, Yuanyuan Yu, Luyao Wang, Duoli Xie, Zhenlin Zhang, Lin Chen, Aiping Lu, Ge Zhang, Fangfei Li

**Affiliations:** ^1^Law Sau Fai Institute for Advancing Translational Medicine in Bone and Joint Diseases, School of Chinese Medicine, Hong Kong Baptist University, Kowloon Tsai, Hong Kong; ^2^Institute of Integrated Bioinfomedicine and Translational Science, School of Chinese Medicine, Hong Kong Baptist University, Kowloon Tsai, Hong Kong; ^3^Institute of Precision Medicine and Innovative Drug Discovery, HKBU Institute for Research and Continuing Education, Shenzhen, China; ^4^Jiangsu Key Laboratory of Xenotransplantation, School of Basic Medical Science, Nanjing Medical University, Nanjing, China; ^5^Shanghai Clinical Research Center of Bone Disease, Department of Osteoporosis and Bone Disease, Shanghai Jiao Tong University Affiliated Sixth People’s Hospital, Shanghai, China; ^6^Department of Wound Repair and Rehabilitation Medicine, State Key Laboratory of Trauma, Burns and Combined Injury, Trauma Center, Research Institute of Surgery, Daping Hospital, Army Medical University, Chongqing, China; ^7^Institute of Basic Research in Clinical Medicine, China Academy of Chinese Medical Sciences, Beijing, China; ^8^Institute of Arthritis Research, Shanghai Academy of Chinese Medical Sciences, Shanghai, China

**Keywords:** disorders of calcium and phosphorus metabolism, PTH-1, 25(OH)_2_D-FGF23 axis, proteomics, metabolomics, biomarkers

## Abstract

Since calcium and phosphorus play vital roles in a multitude of physiologic systems, disorders of calcium and phosphorus metabolism always lead to severe consequences such as skeletal-related and cardiovascular morbidity, or even life-threatening. Physiologically, the maintenance of calcium and phosphorus homeostasis is achieved via a variety of concerted actions of hormones such as parathyroid hormone (PTH), vitamin D, and fibroblast growth factor (FGF23), which could be regulated mainly at three organs, the intestine, kidney, and bone. Disruption of any organ or factor might lead to disorders of calcium and phosphorus metabolism. Currently, lacking of accurate diagnostic approaches and unknown molecular basis of pathophysiology will result in patients being unable to receive a precise diagnosis and personalized treatment timely. Therefore, it is urgent to identify early diagnostic biomarkers and develop therapeutic strategies. Fortunately, proteomics and metabolomics offer promising tools to discover novel indicators and further understanding of pathological mechanisms. Therefore, in this review, we will give a systematic introduction on PTH-1,25(OH)_2_D-FGF23 axis in the disorders of calcium and phosphorus metabolism, diagnostic biomarkers identified, and potential altered metabolic pathways involved.

## Introduction

Disorders of calcium and phosphorus metabolism occur when calcium and phosphorus levels in the body deviate from basal condition, which could be classified into hypercalcemia, hypocalcemia, hyperphosphatemia, and hypophosphatemia. In the view of biological characteristics of this disease, changes in calcium, phosphate, alkaline phosphatase (ALP) and hormone levels such as parathyroid hormone (PTH), vitamin D, and fibroblast growth factor (FGF23) will eventually occur, which could severely influence the life quality of patients and even indirectly shorten life expectancy by inducing growth retardation, osteomalacia, skeletal and dental anomalies, muscle spasms, nephrolithiasis, hypocalcemia convulsions, etc., since calcium and phosphorus are essential components in bone mineralization (Takeda et al., 2004; van Abel et al., 2005; Civitelli and Ziambaras, 2011). In addition, they could also play critical roles in a multitude of physiological processes. For instance, calcium is involved in hormones secretion, the blood-clotting, and the nerve excitement, while phosphorus is required for energy metabolism, cell signaling, and the stabilization of phospholipid content on cell membrane (Takeda et al., 2004; Lambers et al., 2006). Therefore, the regulation of calcium and phosphate ions within narrow limits is critical in maintaining normal physiological activity. It tightly relies on three target organs: intestine, kidney, and bone.

Accumulating evidence suggests that a variety of concerted actions of hormones, including parathyroid hormone (PTH), vitamin D, and fibroblast growth factor (FGF23) associate with nearly each target organ in maintaining the calcium and phosphorus homeostasis (Renkema et al., 2008; Peacock, 2010). On the one hand, when the ionized calcium concentration rises, the thyroid gland increases the secretion of calcitonin, a polypeptide hormone that could reduce the flow of PTH (Talmage et al., 1980; Austin and Heath, 1981). Therefore, the resulting down-regulated PTH inhibits removal of calcium from the bone, increases the loss of calcium in the urine and reduces intestinal calcium absorption through inhibiting the production of vitamin D active form 1,25(OH)_2_D, the chief mediator for calcium absorption at intestine (Khundmiri et al., 2016). Notably, PTH has a more significant effect on enhancing the excretion of phosphate ions in the urine. Since phosphates could combine with calcium ions to form insoluble salts, and then remove them from the serum exchangeable pool. As a consequence, reduced PTH corrects the high calcium concentration toward the average level. On the other hand, the reduction of ionized calcium concentration could be detected by the calcium-sensing receptor (CaSR) located in parathyroid cells, which will improve the rapid release of PTH accordingly. The up-regulated PTH and 1,25(OH)_2_D corrects ionized calcium concentration through the same route. Also, FGF23 could decrease the phosphate levels through reducing the renal phosphate reabsorption and intestinal absorption by inhibiting the production of 1,25(OH)_2_D (Shimada et al., 2004; Martin et al., 2012; Razali et al., 2015).

Based on the detailed understanding of calcium and phosphorus metabolism, we could better explore many genetic or non-hereditary factors that will cause disorders of calcium and phosphorus metabolism via impairing the functions of organs or concentrations of the related hormones, respectively. For example, hyperparathyroidism and hypoparathyroidism could induce the imbalance of calcium and phosphorus via influencing the levels of PTH. Vitamin D deficiency or hereditary factors resulting in changes of 1,25(OH)_2_D, PTH, and FGF23 levels could cause genetic or non-hereditary rickets. Additionally, malignancy is also a common cause for disorders of calcium and phosphorus metabolism via direct invasion of bone in Local Osteolytic Hypercalcemia (LOH), enhancing the flow of parathyroid hormone-related protein (PTHrP), which has the similar function with PTH, or increased synthesis of 1,25(OH)_2_D in various lymphoid tumors (Guise et al., 1996). Acute or chronic kidney disease could disrupt the phosphorus balance through phosphate excretion failure and impair calcium metabolism through a loss of vitamin synthesis capacity. Although there are some clinical methods identified to diagnose the disorders of calcium and phosphorus metabolism, including blood tests, bone density scan, radiography, and bone biopsy, they are not universally applicable due to great trauma, high price, and low specificity (Nickolas and Jamal, 2015). Therefore, it is necessary to explore novel diagnostic biomarkers valuable to detect this disease.

For the in-depth understanding of the disorders of calcium and phosphorus metabolism, omics help a lot, especially proteomics and metabolomics. Proteomics generally refers to analysis of a wide range of proteins, which vary with time and different states the organism undergoes (Anderson and Anderson, 1998; Blackstock and Weir, 1999). While metabolomics, an emerging technology to study metabolites, the small molecule substrates, provides a direct readout of organism state (Nicholson, 2006). An essential focus of proteomics and metabolomics is the identification of novel biomarkers, which could detect disease quickly. For instance, immunoglobulin heavy constant alpha 2 was identified by Kang et al. (2012) as a potential breast cancer metastasis indicator for diagnosis, also, Nicholson et al. (2011) suggested the branched-chain amino acids such as isoleucine might be a valuable biomarker associated with Type 2 diabetes. Another focus is the elucidation of pathogenesis and the discovery of personalized and precision treatment relies on the molecular characteristics associated with a disease (Vaidyanathan, 2012). For example, Jamshidi et al. (2011) found nitric oxide synthase pathway altered significantly in a patient with Hereditary Hemorrhagic Telangiectasia (HHT) by metabolomics studies. Accordingly, the patient’ health has been improved by treating with bevacizumab, an anti-VEGF drug that associated with nitric oxide production. We will also provide current examples of proteomics and metabolomics applications in the disorders of calcium and phosphorus metabolism at the end of this review.

## Calcium Metabolism

Calcium is the most abundant ion in the body, which plays a pivotal role in cell membrane function and intracellular signaling. More than 99% of total calcium is located in bone, acting structurally as supporting material in the form of calcium hydroxyapatite [Ca_10_(PO_4_)_6_(OH)_2_] (Peacock, 2010). In contrast, less than 1% disperses in extracellular fluids, and intracellular calcium levels are extremely low. Calcium in extracellular fluids, intracellular fluids, and bone are balanced by the calcium absorbed in the diet at the small intestine. Calcium exchange between extracellular fluids and bone is an essential dynamic part of bone remodeling via a rapidly exchangeable pool. Urinary excretion is also involved in achieving the calcium homeostasis (Parfitt, 1976a,b; Kumar, 1991).

Calcium in extracellular fluids is present in three fractions. About 40% bound to proteins in the blood, primarily albumin, which could not be filterable by the kidney, whereas an additional 10% of calcium circulates as soluble components combing with various organic anions. The remaining, the most critical form of calcium in extracellular fluids exists as an active free ionized fraction, which determines the physiological effect of calcium (Bushinsky and Monk, 1998), because it interacts directly with cell membranes, with calcium channels, and with the calcium-sensing receptor (CaSR).

Calcium-sensing receptor is abundantly in the parathyroid cells, where it represents the molecular mechanism by which parathyroid cells detect a tiny reduction of ionized calcium concentration and modulate the rapid release of PTH accordingly (Brown et al., 1993; Brown et al., 1995; Hebert, 1996). Briefly, decreased plasma calcium concentration will inactivate CaSR, leading to increased flow of PTH, which has a direct effect on the renal calcium reabsorption and bone absorption through the interaction with the receptorPTH1R (van Abel et al., 2005; Peacock, 2010). Persistent changes in PTH concentration will also enhance intestinal calcium absorption through promoting 25-hydroxyvitamin D3-1α-hydroxylase activity to manufacture vitamin D active form 1,25(OH)_2_D, or calcitriol, the central mediator for calcium absorption at intestine (Burnett-Bowie et al., 2009). Physiological concentration of 1,25(OH)_2_D could enter target cells to interact with its nuclear receptor VDR. The complex then heterodimerizes with the retinoic acid × receptor (RXR), which subsequently interacts with the vitamin D response element (VDRE) on the target gene to control its expression (Hendy et al., 2006; Christakos et al., 2016). Note that although the 1,25(OH)_2_D manufacture in the kidney could be promoted by PTH, itself plays an inhibitory role in the synthesis and secretion of PTH, which might be explained by a feedback loop (Demay et al., 1992). Conversely, when the calcium levels rise, the thyroid gland will increase the flow of calcitonin, another polypeptide hormone, which could reduce the secretion of PTH, resulting in the opposite direction (Talmage et al., 1980; Austin and Heath, 1981).

Intestinal handling of calcium is tightly regulated by 1,25(OH)_2_D and occurs in a passive route or an energy-dependent transcellular pathway (Pérez et al., 2008). The paracellular route depends on the concentration gradient of calcium along the tight junction in the intestinal epithelium. While, the active pathway for intestinal calcium absorption is subject to rather strict transcriptional control of 1,25(OH)_2_D, which represents a pivotal adaptive mechanism under calcium deficiency.

In the active pathway, the intestinal calcium channels TRPV (Transient receptor potential channels, and “V” for vanilloid), primarily TRPV6 but also TRPV5, are responsible for calcium entry (Hoenderop et al., 1999; Hoenderop et al., 2001). Once absorbed, the transcellular movement of calcium in the cytosol to the opposite side is completed under the help of calbindin, a vitamin D-dependent protein. Then the egress of calcium is mediated by the Na^+^/Ca^+^ exchanger (NCX1) through using energy provided by Ca-ATPase or Ca^+^ pump (PMCA1b) (Pérez et al., 2008). Recently, a study discovered a critical role of a channel kinase, TRPM7 (“M” for melastatin), for mineral homeostasis. Mittermeier et al. (2019) showed that TRPM7 depletion resulted in a sharply decline in calcium concentration, suggesting in addition to the factors discussed above, TRPM7 is also necessary for the massive calcium absorption at the intestine, representing another path that need more in-depth exploration. Therefore, the whole process is carefully controlled by 1,25(OH)_2_D via its properties of gene transcription regulation of the essential proteins including TRPVs, TRPM7, calbindin, PMCA1b, and NCX1 (Malloy et al., 1999; Fleet et al., 2002; Song et al., 2003). The paracellular route might also be mediated by 1,25(OH)_2_D partly, but it remains unknown (Chirayath et al., 1998).

In the kidney, approximately 70% will be reabsorbed via a passive route driven by concentration gradient. In contrast, 25–27% will be reabsorbed in the thick ascending limb of the loop of Henle and in the distal convoluted tubule and collecting tubule by active pathway, mirroring the intestinal calcium absorption described previously (Hoenderop et al., 2005; van de Graaf et al., 2007; de Groot et al., 2008; Renkema et al., 2008). Note that PTH has a more significant influence on the urinary excretion of phosphate ions than calcium (Blaine et al., 2015). Since phosphates could combine with calcium ions to form insoluble complexes, more phosphate removal will ultimately raise the serum ionized calcium levels. It is worth mentioning that the kidney could also influence the plasma calcium concentration through processing vitamin D into calcitriol under the influence of high PTH levels.

Since bone contains 99% of the total calcium, it serves as an essential storage organ for calcium. It is a complicated and dynamic tissue mediated by bone-absorptive osteoclasts and bone-forming osteoblasts (Martin and Sims, 2005; Florencio-Silva et al., 2015). Studies have shown that calcium removal from bone is not only regulated by PTH and 1,25(OH)_2_D, under whose influence on the receptor activator of NF-κB ligand (RANKL) to promote maturation of osteoclasts (Boyle et al., 2003; Hamann and Lane, 2006; Bilezikian, 2008; Boyce and Xing, 2008; Blaine et al., 2015), but also could be directly affected by changes in local calcium concentration independent of the above factors, although the molecular basis is not well studied (Dvorak and Riccardi, 2004).

## Phosphorus Metabolism

Phosphorus is critical for many normal biological activities, including muscle contraction, cell signaling, and stabilization of membranes. Similar to calcium, phosphorus is also found predominantly in mineralized bone in the form of hydroxyapatite, approximately 10% exists in soft tissues, and the remaining 2–3% circulates in the extracellular fluids, constituting a phosphate pool that could be rapid exchangeable. Plasma phosphorus balance is the result of phosphate uptake at intestine and reabsorption in the kidney. Both two approaches are regulated by sodium-dependent phosphate cotransporters (Na^+^/Pi-cotransporters) belonging to the *SLC34* (NaPi-IIa, NaPi-IIc, and NaPi-IIb) or *SLC20* gene families of solute carriers, although the latter has been proved to contributes less (Virkki et al., 2007; Biber et al., 2013; Wagner et al., 2014).

Intestinal phosphorus absorption occurs predominantly in the passive paracellular pathway through the luminal concentration of phosphorus. A small energy-dependent transcellular route occurs through NaPi-IIb across the apical brush-border membrane. When the phosphorus concentration decreases, 1α-hydroxylase in the kidney will be activated to manufacture 1,25(OH)_2_D (Hughes et al., 1975), which could increase the expression levels of NaPi-IIb, resulting in enhanced phosphorus absorption at intestine (Katai et al., 1999; Xu et al., 2002).

Phosphate reabsorption in the kidney is mediated primarily by NaPi-IIa channels, while NaPi-IIc play a lesser role (Beck et al., 1998). There is a variety of hormones that influence the renal handling of phosphorus through NaPi-II cotransporters. The most well studied is PTH, which could reduce protein trafficking of the NaPi-II cotransporters to the membrane via endocytosis (Traebert et al., 2000a,b; Yang et al., 2004). Fibroblast growth factor 23 (FGF23), another critical hormone that reduces the phosphorus levels, also has been shown to decrease NaPi-II cotransporters expression in the proximal tubule (Schiavi, 2006; Razzaque and Lanske, 2007; Farrow et al., 2009). Also, the phosphorus-reducing effect of FGF23 could also be achieved via its inhibitory role on 1,25(OH)_2_D (Shimada et al., 2004; Martin et al., 2012; Razali et al., 2015). It is noteworthy that increased 1,25(OH)_2_D concentration appears to promote FGF23 release, suggesting that the inhibitory role of FGF23 on 1,25(OH)_2_D is likely part of a negative feedback loop on phosphorus balance (Kolek et al., 2005; Masuyama et al., 2006). Intriguingly, FGF23 could also inhibit PTH in an alpha-klotho (KL)-dependent manner (Ben-Dov et al., 2007). However, PTH appears to stimulate the synthesis of FGF23 via mechanisms involving PKA/Wnt pathway (Lavi-Moshayoff et al., 2010).

Under average conditions, adults have a net skeletal phosphorus balance close to zero through adjusting the amount of phosphate absorption and urinary excretion. Several essential factors, including phosphatonins, the phosphate-regulating genes associated with endopeptidase (PHEX), etc., that could contribute to the changes in levels of FGF23, which is involved in vitamin D metabolism, also have been identified as indirect factors participating in phosphorus metabolism in bone (Quarles, 2003). However, the comprehensive understanding of phosphorus metabolism in bone still remains elusive, which needs further investigation.

**FIGURE 1:**
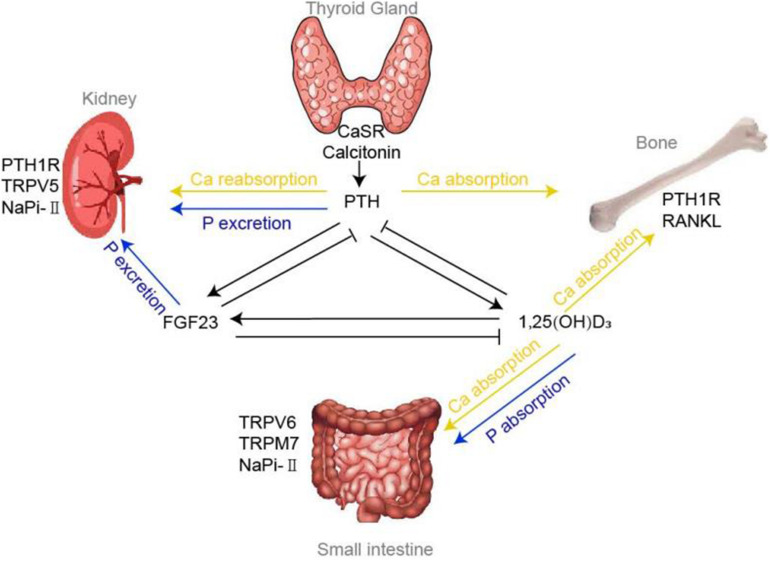
Calcium and phosphorus homeostasis. Regulation of calcium and phosphorus homeostasis tightly relies on a variety of concerted actions of three target organs: intestine, kidney and bone, whose functions are principally regulated by a panel of hormones, including PTH, vitamin D, FGF23 and various factors. Tiny changes in calcium could be sensed by CaSR and Calcitonin located in the thyroid gland, thereby modulating the flow of PTH accordingly. Under the influence of effectors of TRPVs, NaPi-II cotransporters, PTH1R, etc., PTH mediates the absorption and reabsorption of calcium and phosphorus in the bones and kidneys. 1,25(OH)_2_D, the chief mediator for calcium and phosphorus absorption at intestine, could also regulate the bone calcium absorption via RANKL system. FGF23, another critical hormone that promote phosphorus urinary excretion, has been shown to decrease NaPi-II cotransporters expression in the proximal tubule.

In conclusion, maintenance of calcium and phosphate ions within narrow range tightly relies on three organs: intestine, kidney, and bone. And the organs’ functions are principally regulated via several hormones, including PTH, vitamin D, and FGF23. Among them, PTH maintains calcium and phosphorus homeostasis by (1) enhancing the intestinal calcium and phosphate absorption through promoting the manufacture of 1,25(OH)_2_D; (2) increasing phosphorus excretion through its internalization effect on NaPi-II cotransporters and interaction with PTH1R in the kidney; (3) increasing bone absorption. The most important function of vitamin D is to promote the absorption of calcium and phosphorus at intestine. In addition, it could also act on osteoclasts to release calcium ions by promoting RANKL system. FGF23 could reduce phosphorus levels by inhibitory role on NaPi-II cotransporters in the kidney and promote the phosphorus uptake into the bone indirectly. Also, the identification of the vital TRPV, TRPM7, VDR, NaPi cotransporters, and CaSR all have further advanced our understanding of calcium and phosphorus homeostasis and even the disorders of mineral metabolism (shown in Figure 1).

## Disorders of Calcium and Phosphorus Metabolism

### Hypercalcemia

Hypercalcemia occurs in approximately 1 in 500 among the general adult population (Waters, 2009). Malignancy and hyperparathyroidism are the two most common causes (Bilezikian and Silverberg, 2004; Stewart, 2005). Up to 20–30% of cancer patients are reported to have symptoms of hypercalcemia due to (1) direct invasion of bone in Local Osteolytic Hypercalcemia (LOH); (2) Humoral hyperkalemia of malignancy (HHM); Or (3) increased synthesis of 1,25(OH)_2_D (Insogna and Broadus, 1987). In patients with LOH, the hypercalcemia results from a variety of osteoclast activating factors, which are responsible for the significant increase in osteoclast absorption (Roodman, 2004), while HHM is caused by considerable flow of parathyroid hormone-related protein (PTHrP) derived from malignant cells (Guise et al., 1996). Although PTHrP bears similarity to PTH only in the initial eight amino acids, its capacity to bind to the PTH receptor is basically the same, which promotes bone absorption and increases renal reabsorption of calcium (Stewart et al., 1982; Burtis et al., 1988). Various lymphoid tumors, the most well-known Hodgkin’s lymphoma, have been shown to promote intestinal absorption and renal reabsorption of calcium through secreting vast amounts of 1,25(OH)_2_D (Seymour et al., 1994).

Hyperparathyroidism could also be classified into three categories: (1) primary hyperparathyroidism, which is primarily caused by adenomas of parathyroid glands; (2) secondary hyperparathyroidism, which is caused by resultant hyperplasia of parathyroid glands in response to hypocalcemia; (3) tertiary hyperparathyroidism, a term used to describe progressive stage of secondary hyperparathyroidism along with unresponsive flow of PTH. Patients with hyperparathyroidism will show increased synthesis of 1,25(OH)_2_D as a subsequent result of the elevation of PTH levels. Therefore, the promotion of renal reabsorption of calcium by PTH and increased osteoclast and intestinal calcium absorption by 1,25(OH)_2_D lead to the occurrence of hypercalcemia.

Also, patients with Familial hypocalciuric hypercalcemia (FHH), a rare inherited disease with mutation in CaSR, could also result in hypercalcemia because the inactive CaSR in the parathyroid gland will lead to a slight elevation of PTH, similar to the hyperparathyroidism discussed above (Carroll and Schade, 2003). Furthermore, sarcoidosis, a multisystem granulomatous disorder, the cause of which is still not elucidated, could also result in hypercalcemia because of the elevated levels of 1,25(OH)_2_D (Iannuzzi et al., 2007).

### Hypocalcemia

Also, the common causes of hypocalcemia are PTH related, or vitamin D described (Zivin et al., 2001). Contrary to hyperparathyroidism, hypoparathyroidism, which refers to the impaired secretion of PTH due to the irreversible parathyroidectomy, radiation damage, and congenital disabilities, is the leading cause of chronic hypocalcemia. A similar disease term called pseudohypoparathyroidism (PsHP), showing resistant to PTH, is also characterized by hypocalcemia. In addition, the lack of vitamin D caused by UV exposure deficiency or kidney dysfunction, could also result in hypocalcemia (Cooper and Gittoes, 2008).

### Hyperphosphatemia

Because the organs that regulate phosphorus balance are principally the intestine, kidney, and some other tissues, hyperphosphatemia will occur when intestinal absorption rises, renal excretion in acute or chronic kidney disease reduces, or tissue release of phosphorus to extracellular fluids enhances. It is noteworthy that in addition to the hypocalcemia resulting from kidney disease because of impaired vitamin D synthesis, hyperphosphatemia could also occur in chronic kidney disease (CKD) caused by the phosphorus excretion failure. Since phosphates could combine with calcium ions to form insoluble complexes, hyperphosphatemia-induced calcium deposits in the soft tissues will ultimately lead to abnormalities in bone morphology such as bone mineralization and calcification of blood vessels (Fukagawa et al., 2017). At the 2006 Spanish Mineral Metabolism and Bone Disease Conference, the Kidney Disease Improving Global Outcomes (KDIGO) officially named it as chronic kidney disease-mineral and bone disorder (CKD-MBD), while the hyperphosphatemia is an essential biochemical feature (Khwaja, 2010; Bover et al., 2016; Ketteler et al., 2017). Hypoparathyroidism-related hyperphosphatemia refers to the low levels of PTH, which typically promotes phosphate excretion. Therefore, PTH insufficiency could result in more phosphate remaining in the blood. Also, increased tissue release of phosphorus could also occur in tumor lysis syndrome, rhabdomyolysis, hemolysis, hyperthermia, acute leukemia, etc. (Voelkl et al., 2019).

### Hypophosphatemia

Similar to the factors causing hyperphosphatemia, hypophosphatemia will occur when there is inadequate phosphorus intake at intestine or excessive renal wasting resulting from rickets or Fanconi’s syndrome.

Rickets is a metabolic bone disease, which refers to inadequate mineralization of growing bones caused by abnormal metabolism of calcium, phosphorus, and/or vitamin D. It could be divided into nutritional rickets and hereditary rickets due to different pathogenesis.

Hereditary rickets could be classified into two categories due to different types of gene variations: (1) vitamin D-dependent rickets (Miller, 2017); and (2) hereditary hypophosphatemic rickets (HP) (Beck-Nielsen et al., 2012). Moreover, vitamin D-dependent rickets might be further classified into type 1A (VDDR1A) and type 1B (VDDR1B) associated with vitamin D synthesis, and type 2A (VDDR2A) and type 2B (VDDR2B) pertaining to vitamin D receptor activity in more detail. Therefore, its typical biochemical characteristics are hypocalcemia and hypophosphatemia caused by vitamin D deficiency. VDDR1A is due to the deletion of 1-alpha hydroxylase enzyme caused by mutations in the *CPY27B1* gene. While VDDR1B results from the deletion of first-step enzyme, 25-hydroxylase, caused by the mutations in the *CYP2R1* gene. Due to variations in the vitamin D receptor (*VDR*) gene, VDDR2A shows resistance to 1,25(OH)_2_D. However, unlike VDDR2A, the function of VDR is normal in VDDR2B, which prevents the binding of VDR-RXR heterodimer to VDR element (VDRE), resulting in abnormal vitamin D transcriptional function (Fraser et al., 1973).

Hereditary hypophosphatemic rickets (HR) is a rare group of renal phosphate wasting disorders. Hypophosphatemia and average calcium concentration are their typical biochemical characteristics. It is a type of hereditary rickets caused by the variations in phosphoproteins or cotransporters, which are necessary for renal phosphate reabsorption (Guven et al., 2017). So far, a panel of genetic causes has been identified. Some genetic mutations lead to increased serum FGF23 concentration (FGF23-dependent HR), while others influence phosphate transporters without affecting serum FGF23 levels (FGF23-independent HR). Although these two types could be distinguished by FGF23 concentration easily, the diseases within the group need to be differentiated by further molecular testing.

X-linked dominant HR (XLDHR) caused by *PHEX* gene mutation is the most common type of FGF23-dependent HR. *PHEX* gene product does not seem to regulate FGF23 clearance directly, but to encode a membrane endopeptidase, which could promote the removal of FGF23 (Rowe, 2012). Therefore, *PHEX* mutation could result in elevated FGF23, which is unable to degrade. High levels of FGF23 could also occur in Autosomal Dominant Hypophosphatemic Rickets (ADHR), which is caused by the inactive mutation in the proteolytic cleavage domain (RXXR cleavage motif) in FGF23, therefore the variation renders FGF23 resistant to proteolytic cleavage (Shimada et al., 2002). Also, increasing serum FGF23 concentration could also result from the Autosomal Recessive Hypophosphatemic Rickets (ARHR), including 1) Autosomal Recessive Hypophosphatemic Rickets Type 1 (ARHR1), which is caused by inactive mutation in the dentin matrix acidic phosphoprotein (DMP1) gene acting on the inhibition of FGF23 expression, and 2) Autosomal Recessive Hypophosphatemic Rickets Type 2 (ARHR2), which is caused by ectonucleotide pyrophosphatase/phosphodiesterase 1 (ENPP1) inactive mutation (Lorenz-Depiereux et al., 2006). Furthermore, a rare disease termed Hypophosphatemic Rickets with Hyperparathyroidism caused by a balanced translocation adjacent to the KL gene, whose product is FGF23’s necessary cofactor alpha-Klotho, could lead to an increase of circulating alpha-Klotho. As a result, since alpha-Klotho could stimulate the expression of FGF23, it might explain the up-regulation of FGF23 (Brownstein et al., 2008). Other rare genetic diseases involving osteoglophonic dysplasia, McCune–Albright syndrome, Raine syndrome, opsismodysplasia, etc., could also cause hypophosphatemia through influencing FGF23 concentration (Acar et al., 2017).

In addition to changes in FGF23 levels, cotransporters and PTH receptor variations could also induce hypophosphatemia via disrupted phosphorus reabsorption metabolism. Hereditary Hypophosphatemic Rickets with Hypercalciuria (HHRH) and Hypophosphatemic Rickets with Nephrolithiasis and Osteoporosis Type 1 (NPHLOP1), which are caused by the inactive mutations in *SLC34A3* (also known as NaPi-IIc) and *SLC34A1* (also known as NaPi-IIa) respectively, could result in hypophosphatemia through the abnormal renal reabsorption process (Tencza et al., 2009). Hypophosphatemic Rickets with Nephrolithiasis and Osteoporosis Type 2 (NPHLOP2), caused by an inactive mutation of *SLC9A3R1* (also known as NHERF1), which encodes an adaptor protein that regulates several G protein-coupled receptors including PTH1R. Moreover, Dent Disease is the result of mutations in the *CLCN5* or *OCRL* gene, which is characterized by renal dysfunction, thereby eventually resulting in calcium and phosphorus disorders (Devuyst and Thakker, 2010).

In addition to hereditary rickets, nutritional rickets is also a serious global public health problem, which refers to abnormal bone mineralization caused by vitamin D deficiency. Furthermore, both genetic and acquired Fanconi’s syndrome could also lead to renal phosphate wasting in urine due to defects in the proximal tubule (Izzedine et al., 2003). The classification of disorders of calcium and phosphorus metabolism is summarized in Table 1.

**TABLE 1:** Classification of disorders of calcium and phosphorus metabolism.

Type	Causes
Hypercalcemia	Malignancy Hyperparathyroidism Familial hypocalciuric hypercalcemia (FHH) Sarcoidosis, etc.
Hypocalcemia	Hypoparathyroidism Pseudohypoparathyroidism (PsHP) Vitamin D deficiency
Hyperphosphatemia	Kidney disease Hypoparathyroidism Tumor lysis syndrome Rhabdomyolysis Hemolysis Hyperthermia Acute leukemia, etc.
Hypophosphatemia	Hereditary rickets: Vitamin D-dependent rickets: (VDDR1A, VDDR1B, VDDR2A, VDDR2B). Hereditary hypophosphatemic rickets: (XLDHR, ADHR, ARHR, Hypophosphatemic Rickets with Hyperparathyroidism, McCune-Albright syndrome, Raine syndrome, Opsismodysplasia, HHRH, NPHLOP1, NPHLOP2, Dent Disease) Nutritional rickets Fanconi’s syndrome

## Application of Proteomics/Metabolomics in Disorders of Calcium and Phosphorus Metabolism

Proteomics and Metabolomics/Metabonomics are essential branches of system biology. Although all proteins are based on DNA, post translational modifications render gene analysis alone is impossible to define the abundance of proteins. The proteome consists of all proteins present in particular cell types or tissues, while the metabolome consists of small molecules known as metabolites (Blackstock and Weir, 1999; Hollywood et al., 2006). They are technologies that comprehensively characterize the protein and metabolic levels of cells or organisms to study the roles of proteins and the metabolic profiles in biological and pathological systems (Nicholson et al., 1999). Therefore, they are promising approaches to identify biomarkers or unmask perturbations and signal pathways associated with disease through available biological samples, such as urine (Want et al., 2010; Cheng et al., 2012), plasma, etc. (Qiu et al., 2009). Also, a rapidly growing body of proteomics and metabolomics related work has promoted the development of precision medicine and has been widely concerned (Suhre et al., 2011). To illustrate the current status of the applications of proteomics and metabolomics in disorders of calcium and phosphorus metabolism, we discuss here the advances that used these technologies to investigate novel biomarkers and disturbed pathways related with this disease.

## Proteomics/Metabolomics Studies on Primary Hyperparathyroidism

Primary hyperparathyroidism (PHPT) is characterized by hypercalcemia and abnormal excessive flow of PTH. To unveil the pathophysiology of PHPT, molecular details about parathyroid hyperplasia and adenoma, two similar causes contributing to the PHPT, must be revealed. Therefore, Akpinar et al. (2017) performed a study to differentiate these two diseases through comparative proteomics. Forty novel dysregulated proteins of interest were identified, of which 14 were up-regulated in hyperplasia and 26 were overexpressed in adenoma. It is worth mentioning that most of the up-regulated proteins in hyperplasia identified are mitochondrial proteins, involving cytochrome b-c1 complex subunit 1, enoyl-CoA hydratase, 60 kDa heat shock protein, etc., suggesting the importance of abnormal mitochondrial activity in the pathogenesis of PHPT. Although this research could not create a biomarker panel, the changes in mitochondrial proteins expression yield important direction for future studies to differentiate parathyroid hyperplasia and adenoma, which remained a significant challenge to dissolve (Akpinar et al., 2017). Although parathyroid adenomas account for approximately 85% cases of PHPT (Carling, 2001), its pathogenesis is only partially understood. Varshney et al. (2014) showed that 15 differentially expressed proteins involving in diverse cellular functions were found between adenomatous and healthy parathyroid tissues through proteomics. Although functional categorization of dysregulated proteins did not focus on a certain signaling, the services of the identified proteins associate with a massive number of pathways, including the regulation of programed cell death, transcription, and signal transduction, etc. Among them, ATP synthase subunit d protein is also reported in the research by Akpinar et al. (2017). However, majority of the identified proteins are different with the earlier study by Giusti et al. (2011) which might be explained by the different disease severity and ethnicity (Asian Indian vs. Caucasian), that needs further validation. Therefore, the characterized proteins might provide new directions for the in-depth exploration of molecular details of the disease and novel therapeutic options that target at the differentially expressed proteins (Varshney et al., 2014).

On the one hand, evidence shows that parathyroid adenomas might consist of chief cells, oxyphilic cells, or a mixture of both cells (Williams, 1974). To differentiate different cell types within parathyroid adenomas, Lu et al. (2018) identified a series of proteins that altered significantly between chief and oxyphilic cell adenomas, and p53 pathway might be involved through proteomics analysis, since the higher expression of LMO3 and S100B in oxyphilic cells, which could inhibit the transcription of the *TP53* gene. Intriguingly, lower expression of nuclear receptor TR4 was reported in oxyphilic cells compared to chief cells. Evidence has shown that mitochondria accumulation was observed in TR4–/– mice, implicating decreased TR4 level might contribute to the oxyphilic cells transformation and formation, which is characterized by high mitochondrial content. In addition, compared to cytoplasm and nuclei subcellular location of TR4 in normal parathyroid, it mainly localized in the nuclei of adenomas, implying different roles of TR4 in adenomas that remains elusive, which needs further investigation. On the other hand, PHPT could also be classified into two different groups based on their relative responsiveness to ambient calcium. Koh et al. (2018) identified a set of candidate genes expressed differently between the calcium-sensitive and calcium-resistant parathyroid tumors by RNA seq and proteomics analysis. Notably, based on the results of studies by Akpinar et al. (2017) elevated expression of mitochondrial genes such as *COX7B*, *CYCS*, and *APT5G3* were observed in calcium-resistant group, providing a potential relationship between calcium-resistant (mitochondria-rich) and hyperplasia, which needs additional research to address. In calcium-sensitive group, ECM and trafficking genes, *TNXB* and *LIN7A*, which are necessary for CaSR-mediated signaling, showed significant high levels. Hence, the results suggest that the different PHPT clinical presentation, provenance and outcome might be caused by different molecular basis, which remains unknown (Koh et al., 2018).

A single gland disease (SGD), which is caused by a single parathyroid adenoma, and multiglandular disease (MGD), which is recognized with multiple adenomas or multiglandular hyperplasia, both could result in PHPT; however, there are no biomarkers available to differentiate these two types. Therefore, Battini et al. (2016) aimed to compare the tissue metabolome profiles between SGD and MGD in PHPT to investigate the difference, and eventually identified a multitude of metabolites which could accurately predict SGD from MGD. For instance, compared with MGD, elevated levels of succinate and fumarate were observed in SGD, which involved in the tricarboxylic acid cycle, while a higher expression of glutamate, GSH, and ascorbate serving as antioxidants in MGD. However, due to the limited samples, data should be validated in further research (Battini et al., 2016).

Normocalcemic primary hyperparathyroidism (NC-PHPT), the first subclinical phase of PHPT, is a new clinical stage characterized by average serum calcium concentration and elevated PTH levels. In contrast, the second stage of termed hypercalcemic primary hyperparathyroidism (HC-PHPT) is typically characterized by a hypercalcemic phase. Notably, emerging evidence has shown that PHPT is typically accompanied by cardiovascular morbidity (Lundgren et al., 2001). However, it is unclear whether NC-PHPT and HC-PHPT share the same risk factors associated with cardiovascular events. Recently, Yener Ozturk et al. (2016) suggested that patients with NC-PHPT have similar metabolome profiles compared with the HC-PHPT group. Therefore, due to the unknown molecular mechanism, NC-PHPT should be further explored in the future for valuable early diagnosis (Yener Ozturk et al., 2016). The above differentially expressed proteins, metabolites, and involving metabolic pathways are shown in Table 2.

**TABLE 2:** Proteomics/Metabolomics studies on PHPT.

Proteins/metabolites	Comparision	Biological process	References
Nucleophosmin, peroxiredoxin-2, Bcl-2-like protein-10, DNAJC2 protein (Hsp40), ATP synthase subunit day, aconitase 2, elongation factor Tu (p43), phosphatidylinositol transfer protein, MED26 protein (isoform-2), fibronectin type III, vacuole membrane protein 1 (HSPC292), prohibitin, nuclear receptor binding factor 2, zinc finger protein 394, Ras-related protein Rab-7b	Parathyroid adenomas vs. normal	Regulation of programed cell death cell organization and transcription, miscellaneous proteins, Ras protein signal transduction	Varshney et al., 2014
Nuclear receptor subfamily 2 group C member 2 (TR4), LIM domain only protein 3 (LMO3), calcium-binding protein B (S100B)	Oxyphilic cell vs. chief cell in parathyroid adenoma	p53 pathway	Lu et al., 2018
Ubiquinone oxidoreductase core subunit V1, cytochrome C oxidase subunit 7B, cytochrome C, ATP synthase, lin-7 homolog A, tenascin, etc.	Calcium-sensitive vs. calcium-resistant parathyroid tumors	Redox metabolism, GPCR trafficking, GPCR signal transduction, PI3 kinase signaling	Koh et al., 2018
Phosphorylcholine, choline, glycerophosphocholine, fumarate, succinate, lactate, glucose, glutamine, ascorbate, etc.	Single gland disease vs. multiglandular disease in PHPT	Tricarboxylic acid cycle	Battini et al., 2016
60 kDa heat shock protein, ubiquitin carboxyl-termial hydrolase, lamin-A/C, heterogeneous nuclear ribonucleo-proteins A2/B1, etc.	Parathyroid hyperplasia vs. adenoma	Energy metabolism, redox metabolism, protein folding etc.	Akpinar et al., 2017

## Proteomics/Metabolomics Studies on CKD-MBD

Based on the status of bone turnover, CKD-MBD could be classified into two groups: (1) High Turnover Bone Disease (HTBD), which is followed by secondary hyperparathyroidism; and (2) Low Turnover Bone Disease (LTBD), which is associated with osteoporosis. In addition, based on evidence suggesting that adipose tissue calcification might originate from the differentiation of stem cells, the exploration of stem cells extracted from adipose tissue of different bone status might provide important clues for the soft tissue calcification mechanism. Recently, a proteomic study performed by Kasap et al. (2015) identified a total of 32 regulated proteins in stem cells and analyzed the potential relationship between different proteins and bone turnover status, suggesting their essential roles in distinguishing HTBD with LTBD. For example, two structural proteins, F-acting-capping protein and keratin, and type 1 cytoskeleton 10, attract our attention due to their significant abundances in HTBD compared to LTBD, indicating the novel roles in determining the bone turnover status. Although the exact functions of them in stem cells are poorly understood, the data offers important source for further discoveries (Kasap et al., 2015).

Although CKD-MBD patients have a considerable mortality and disability rate, the current biomedical predicators are lagging and not sensitive enough. Therefore, according to the guidelines that clinicians should focus more on serum biochemical indicators for diagnosis, which is given by the Kidney Disease Improving Global Outcomes (KDIGO), it is crucial to identify novel early diagnostic biomarkers valuable. Recently, Wu et al. (2015) have identified a set of metabolic biomarkers for CKD-MBD by employing metabolomics. After validating the potential biomarkers, a total of 32 potential biomarkers were identified (Wu et al., 2015). For example, elevated dopamine glucuronide is a promising biomarker for onset of CKD-MBD because dopamine level is positively correlated with phosphate concentration, the high level of which is a typical characteristic of CKD-MBD. In addition, up-regulated glycylprolylhydroxyproline and glutaminyl-hydroxyproline are metabolic products of hydroxyproline, which could act as a bone biomarker that also may be a potential biomarker for CKD-MBD. However, the substrates of TCA cycle intermediates, glutamyl-glutamate and *N*-acetylaspartylglutamic acid, could not be valuable biomarkers because they are involved in a variety of diseases and not specific. Furthermore, the results suggest that metabolic pathway patterns also shift, involving routes of protein synthesis, amino acid metabolism, and steroid hormone metabolism in addition to the established metabolic pathways associated with phosphorus, calcium, PTH, and vitamin D (Levin et al., 2007), with evident promise for better understanding of the pathogenesis in CKD-MBD.

Another similar article performed by Shen et al. (2019) also found various biomarkers correlated to PTH in SHPT. However, in contrast to the above study, which mainly focused on the altered metabolites in CKD-MBD, this study primarily focused on those correlated with PTH. The results indicated that five differential metabolites, allyl isothiocyanate, L-phenylalanine, D-Aspartic acid, indoleacetaldehyde, and D-galactose correlated with PTH could serve as potential biomarkers to diagnose SHPT with good sensitivity and accuracy (Shen et al., 2019). Among them, L-phenylalanine is an activator of CaSR, which shows inhibitory role on PTH secretion, might be an explanation for its diagnostic value as a biomarker for SHPT (Gensure et al., 2003). Additionally, a proteomics study also identified a multitude of mediators of inflammation (IL-6 and TNF-α) and mineral and bone disorders biomarkers (OPG, OPN, OCN, FGF23, and Fetuin-A) as a multiplexed panel to improve early CKD diagnosis. OPG, OPN, and FGF23 could also reflect vascular severity, the leading complication of CKD. Therefore, these studies provide a novel tool for clinical evaluation for CKD staging and therapeutic response (Mihai et al., 2016).

Evidence has shown that the proliferation of oxyphil cell might involve in SHPT development, however, the mechanism is not clarified (Sumida et al., 2011). Therefore, to identify the critical regulatory factors of oxyphil cell proliferation might be helpful to unravel the pathogenesis and therapeutic target of SHPT. Recently, Li et al. (2018) aimed to identify proteins expressed differently in oxyphil compared with chief cells, and eventually found that mitochondrial proteins, consistent with the above results discussed, and metabolisms involving protein synthesis and cell cycle regulation were significantly altered in oxyphil cell. Generally, active vitamin D is used as treatment option but sometime resistance occurs. In this study, down-regulated vitamin D binding protein (DBP) might be a potential explanation in the calcitriol treatment resistance by impairing transportation of vitamin D. The critical MIF-CUL1 axis that associated with ubiquitin mediated proteolysis might participate in the proliferation of oxyphil cell due to their significantly altered expression. The results shed light on mitochondria dysfunction in proliferation mechanisms of oxyphil cells, which accelerates the SHPT progression (Li et al., 2018).

Generally, hyperphosphatemia is a well-known promoter of cardiovascular dysfunction, a severe complication of CKD, which is the leading cause of poor prognosis (Block et al., 2004). Therefore, a metabolomics study aiming to define the molecular basis underlying hyperphosphatemia-induced vascular endothelial dysfunction performed by Zhou et al. (2016) identified 36 metabolites, which were significantly altered in human umbilical vein endothelial cells (HUVECs), involving vast metabolic pathways such as urea cycle, energy metabolism, etc. Among them, ornithine increased significantly. Further study demonstrated that the up-regulation of ornithine might result from the elevated arginase 2, which mediates the hydrolysis of arginine to form ornithine. Therefore, the results point that arginase 2 might serve as a therapeutic target for cardiovascular abnormalities caused by hyperphosphatemia (Zhou et al., 2016). However, further clinical samples analysis from patients to understand the comprehensive changes is necessary due to the limitations of cell models. Proteins and metabolites that expressed differently, and related pathways defined above are summarized in Table 3.

**TABLE 3:** Proteomics/Metabolomics studies on CKD-MBD.

Protein/metabolite	Comparision	Biological process	References
*N*-(1-Deoxy-1-fructosyl) tryptophan, *N*-acetylaspartylglutamic acid, glycylprolylhydroxyproline, (R)-pantothenic acid 4′-*O*-b-D-glucoside, aminohippuric acid, etc.	CKD-MBD vs. normal	Protein synthesis and amino acid metabolism, energy metabolism, and steroid hormone metabolism	Wu et al., 2015
Allyl isothiocyanate, L-phenylalanine, D-Aspartic acid, indoleacetaldehyde, and D-galactose corrected with PTH	SHPT vs. normal	Amino acid metabolism	Shen et al., 2019
Cullin-1, 5′-AMP-activiated protein kinase subunit beta-2, Carnitine palmitoyltransferase 1B, protein, E3 ubiquitin-protein ligase CBL, etc.	Oxyphil cell modules vs. Chief cell modules	Wnt signaling, TGF-β, ubiquitin mediated proteolysis, cell cycle regulation, protein synthesis	Li et al., 2018
Total 36 metabolites, especially ornithine	hyperphosphatemia- associated cardiovascular vs. normal	Urea cycle, arginine-, proline-, metabolism etc.	Zhou et al., 2016
IL-6, TNF-α,OPG,OPN,OCN,FGF-23, Fetuin-A	CKD stage vs. normal	RANK/RANKL/OPF signaling pathway, energy metabolism	Mihai et al., 2016
Vimentin, F-acting-capping protein subunit beta-alfa-1, WD repeat-containing protein 1, prelamin A/C, DnaJ homolog subfamily B member 11, 78kDa glucose-regulated protein, endoplasmin, stress-70 protein, protein disulfide-isomerase A, peroxiredoxin, etc.	High Turnover Bone disease vs. Low Turnover Bone Disease	Redox metabolism, protein biosynthesis degradation, transcription, energy and amino acid metabolism	Kasap et al., 2015

## Proteomics/Metabolomics Studies on Nutritional Rickets

For the diagnosis of nutritional rickets, although there are several serum biochemical tests, such as 25(OH)D, calcium, phosphate, alkaline phosphatase (ALP) and PTH, clinical symptoms and radiography, there are still defects in sensitivityand specificity (Faerk et al., 2002). Therefore, to investigate nutritional rickets-related urinary biomarkers, an unbiasedglobal urinary metabolomics analysis performed by Wang et al. (2014) defined a panel of altered metabolic profiles associated with nutritional rickets. 31 differentially expressed proteins were identified, and five candidate biomarkers for clinical diagnosis were screened. To further improve accuracy, a combination of several biomarkers was employed, such as phosphate and sebacic acid, which could predict nutritional rickets with high sensitivity. Therefore, based on the pathway analysis of biomarkers, the urinary metabolomics analysis gives new insights into the pathophysiology of nutritional rickets (Wang et al., 2014).

## Discussion and Future Perspectives

Since calcium and phosphorus metabolism play vital roles in a multitude of physiologic systems, disorders of calcium and phosphorus metabolism, as a systemic disease, always lead to destructive consequences such as skeletal-related events or even life-threatening. Therefore, it is necessary to understand the calcium and phosphorus metabolism for early diagnosis and corresponding therapeutic strategies. In this paper, the roles of PTH-1,25(OH)_2_D-FGF23 axis in the calcium and phosphorus metabolism were described. And the pathological causes and various categories of this disease were also clearly discussed.

PTH-1,25(OH)_2_D-FGF23 system is critical in the whole process of calcium and phosphorus metabolism. PTH and FGF23 could mediate calcium and phosphorus concentration within tight limits via a rapidly exchangeable pool between bone and extracellular fluids and urinary mineral excretion. Under the influence of PTH, 1,25(OH)_2_D could promote calcium and phosphorus absorption at intestine. Therefore, disorders of calcium and phosphorus metabolism could be caused by the disturbance on PTH-1,25(OH)_2_D-FGF23 system and each impaired function of related organs.

Based on the detailed understanding of the PTH-1,25(OH)_2_D-FGF23 axis, we could better summarize vast types of genetic or no-hereditary disorders of calcium and phosphorus metabolism. On the one hand, changes in PTH concentration are induced by hyperparathyroidism or hypoparathyroidism. On the other hand, rickets, which is caused by vitamin D deficiency or hereditary factors resulting in disturbed levels of PTH or FGF23, has become a global health problem, especially the Hereditary Diseases/Disorders of Calcium and Phosphorus Metabolism (HDCPM). Their early diagnosis and therapy have become an urgent challenge around the world. Also, malignancy is a common cause for disorders of calcium and phosphorus metabolism via direct invasion of bone or up-regulated 1,25(OH)_2_D, whereas acute or chronic kidney disease could disrupt the mineral homeostasis due to phosphate excretion failure or impaired 1,25(OH)_2_D synthesis capacity.

Proteomics and metabolomics, the comprehensive technologies to study the altered proteins and metabolic pathways by systematically characterizing the changes in the proteome and metabolome profiles in a disease state, play essential roles in identifying biomarkers and elucidation of pathological mechanisms. Some proteomics and metabolomics studies have identified a series of valuable biomarkers and potential signaling pathways associated with disorders of calcium and phosphorus metabolism. For example, studies on PHPT found a set of disturbed proteins and metabolites, which could provide new directions for the molecular basis exploration of this disease and better discrimination of different groups of PHPT. In the search for the biomarkers of CKD-MBD and associated calcification complication, biomarkers that could be used for early diagnosis and altered metabolic pathways have been identified. These studies not only shed light on clinical diagnosis and prognosis for CKD-MBD, but also hint the potential therapeutic targets for the cardiovascular morbidity caused by hyperphosphatemia. For the metabolomics analysis of nutritional rickets, a panel of urinary biomarkers with high accuracy and specificity are defined, which could provide new clues into the pathophysiology of this disease. However, a limited number of samples, cell models, etc. need further validation series, and the exact roles of altered proteins/metabolites and metabolic pathways remains a need for in-depth investigation. Although some studies have applied this technology to biomarkers discovery and exploration of molecular mechanisms, there are still many metabolic pathways and clinical diagnostic biomarkers for disorders of calcium and phosphorus metabolism that have not yet been discovered, especially for Hereditary Diseases/Disorders of Calcium and Phosphorus Metabolism (HDCPM), the most common genetic metabolic diseases in newborns. Currently, the omics study on it is still blank; therefore more in-depth omics studies associated with this disease are required in the future.

## Author Contributions

MS wrote the manuscript. FL and XW helped in revising the manuscript. FL, AL, and GZ supervised the preparation of the manuscript. All authors contributed to the article and approved the submitted version.

## Conflict of Interest

The authors declare that the research was conducted in the absence of any commercial or financial relationships that could be construed as a potential conflict of interest.
